# “Is it time to throw out the weighing scales?” Implicit weight bias among healthcare professionals working in bariatric surgery services and their attitude towards non-weight focused approaches

**DOI:** 10.1016/j.eclinm.2022.101770

**Published:** 2022-12-14

**Authors:** S. Abbott, E. Shuttlewood, S.W. Flint, P. Chesworth, H.M. Parretti

**Affiliations:** a Department of Bariatric Surgery, University Hospitals Coventry and Warwickshire NHS Trust, Clifford Bridge Road, Coventry CV2 2DX, UK; b Research Centre for Intelligent Healthcare, Coventry University, Richard Crossman Building, Coventry CV1 5RW, UK; c School of Psychology, University of Leeds, Leeds, West Yorkshire, UK; d Scaled Insights, Nexus, University of Leeds, Leeds, West Yorkshire, UK; e Patient Advocate, National Bariatric Surgery Register, UK; f Norwich Medical School, University of East Anglia, Norwich, Norfolk NR4 7TJ, UK

**Keywords:** Bariatric surgery, Implicit weight bias, Stigma, Healthcare

## Abstract

**Background:**

People living with overweight or obesity (PLwO) can be stigmatised by healthcare professionals (HCPs). Reducing focus on weight is a proposed strategy to provide less threatening healthcare experiences. Given the lack of research on weight bias within obesity services, this study aimed to explore implicit bias among obesity specialist HCPs and explore views on non-weight focused approaches.

**Methods:**

Obesity specialist HCPs were invited to a webinar, “An exploration of non-weight focused approaches within bariatric services”, held in October 2021. Implicit weight bias was examined using the BiasProof mobile device test, based on the Implicit Association Test. Poll data was analysed descriptively, and qualitative data was analysed using framework analysis.

**Findings:**

82 of the 113 HCPs who attended the webinar consented to contribute data to the study. Over half (51%) had an implicit weight bias against PLwO. Most (90%) agreed/strongly agreed that obesity services are too weight focused and that patients should not be weighed at every appointment (86%). Perceived benefits of taking a non-weight focused approach included patient-led care, reducing stigma and supporting patient wellbeing, while perceived barriers included loss of objectivity, inducing risk and difficulty demonstrating effectiveness.

**Interpretation:**

Our findings indicate that half of obesity specialists HCPs in our sample of 82 providers, who are primarily dieticians and psychologists, have an implicit weight bias against PLwO. HCPs feel that a weight-focused approach within services was a barrier to patient care, but that there is a lack of alternative non-weight focused measures. Further research is needed into substitute outcome measures for clinical practice, also seeking the views of PLwO, and into interventions to address implicit weight bias.

**Funding:**

10.13039/100004331Johnson & Johnson funded the BiasProof licence and publication open access charge.

**Research in context**

**Evidence before this study**

Findings from a recent meta-analysis show a moderate pooled effect (SMD 0.66, 95% CI 0.37–0.96) for healthcare professionals’ exhibition of implicit weight bias against people living with obesity. The authors searched PubMed from inception to 1st August 2022 for published literature using terms “implicit weight bias” “healthcare professional” to locate studies within the obesity specialist healthcare professional population. Only two studies were identified, which were undertaken over a decade ago. These studies examined the direction of implicit weight bias but did not present data at the participant level. Therefore, the prevalence of implicit weight bias among healthcare professionals working in obesity services is unknown.

**Added value of this study**

We examined the prevalence and severity of implicit bias amongst healthcare professionals working in obesity services in the UK using the BiasProof application, based upon the Implicit Association Test. We collected data on attitudes and current practice in relation to weight quantitatively from polls, and qualitatively from written open-ended responses. In our study of 82 UK healthcare professionals working in obesity services, over half (51%) exhibited an implicit weight bias against people living with overweight or obesity. Nearly all participants felt that services were “too weight focused” and while there were thematic advantages to taking a less weight-centric approach, there were also barriers.

**Implications of all the available evidence**

Implicit weight bias impacts the relationship between the healthcare professional and the patient, and the quality of care received. The prevalence of implicit weight bias among healthcare professionals working in obesity services is lower compared to those working in other specialities, but is still unacceptably high. Our study highlights the need for targeted interventions to address underlying implicit weight biases amongst all healthcare professionals, but especially obesity specialists who provide obesity treatment.

## Introduction

There is now a wealth of evidence demonstrating that people living with obesity (PLwO) experience weight stigma and discrimination across many settings, including healthcare.^1^, ^2^, ^3^, ^4^, ^5^

The impact of experiencing weight stigma and discrimination in healthcare may include reduced engagement and reduced healthcare seeking behaviour, as well as leading to worsening health outcomes.^3^^,^^6^ For instance, research has shown that experiences of weight stigma are associated with physiological dysregulation,^7^ leading to a stress response that ultimately results in poorer health outcomes,^6^ greater mortality risk^7^^,^^8^ and is linked to depression and anxiety,^9^ and maladaptive coping responses; manifesting in social isolation, disordered eating behaviours and reduced physical activity.^10^

As such, there have been calls for interventions to reduce weight bias amongst healthcare professionals, including improved education to address misconceptions, stigmatising attitudes, and inaccuracies in healthcare professionals' (HCPs’) beliefs about the causes and treatment of obesity that is not aligned to empirical evidence.^4^^,^^11^ For example, large proportions of HCPs report perceptions that obesity can be prevented and treated solely by a commitment to a healthy lifestyle,^11^ which does not align to the wealth of evidence demonstrating the complex, multifaceted nature of obesity.^12^

Clinical encounters contribute to the formation of HCPs’ beliefs and attitudes^13^ and there is a growing concern that the repetitive and narrow focus on weight as both an access point to services and the primary measure of change might be contributing to, and proliferating, stigmatisation of PLwO within the healthcare setting.^14^ Mandating weight loss as a pre-cursor to access to bariatric surgery is a contrived issue. Many bariatric surgery services withhold surgery to patients who do not meet a target weight loss,^15^^,^^16^ despite the lack of scientific evidence to justify such practice.^17^ The dominant weight-centric paradigm in healthcare services may have unintentional negative consequences, including stigmatisation, and therefore there are calls to move away from a weight-centric to health-focused approach to patient care.^13^

The negative attitudes underlying enacted stigma can be explicit, characteristic of conscious beliefs, or implicit, automatic attitudes that occur outside of one's awareness.^18^ There is compelling evidence that HCPs exhibit significant implicit weight bias against PLwO,^19^ however there is limited research amongst HCPs specialising in obesity.

To our knowledge, only two previous studies have examined implicit weight bias amongst healthcare professionals specialising in obesity: first in 2001^20^ and then in a repeated study in 2013.^21^ In these studies, real-time data was collected from delegates at obesity conferences in North America. The latter and most recent study^21^ found that HCPs exhibited a significant pro-thin, anti-fat implicit bias (p ≤ 0.0001). However, implicit bias scores were not categorised at the participant level and therefore we do not have any data to infer the prevalence of implicit weight bias. Moreover, there is no published literature investigating implicit weight bias among HCPs who work within bariatric surgery specialist services, where treatment is provided for people living with the most clinically severe obesity.^22^

Hence, the aim of our study was to determine the prevalence of implicit weight bias among HCPs working in bariatric surgery services and explore practices and attitudes towards weight and non-weight focused approaches to patient care.

## Methods

### Study setting and participants

In October 2021, the British Metabolic and Obesity Surgery Society (BOMSS) hosted the webinar “An exploration of non-weight focused approaches within bariatric services”, delivered via Zoom videoconferencing software. The webinar was free of charge and open to both BOMSS members and non-members. The webinar delegates were HCPs working with PLwO in obesity services in the United Kingdom. All delegates were invited to take part in this study.

### Data collection

Implicit weight bias was examined using the BiasProof^23^ mobile device test which is based on the brief Implicit Association Test.^24^ The test is comprised of five blocks, where participants are exposed to stimuli in the middle of the mobile device, and they are required to assign the stimuli to the category that it corresponds with, on either the left- or right-hand side of the screen. Participants are asked to assign the stimuli to the categories as quickly as possible. There are four grouping categories: overweight, thinness, pleasant and unpleasant. The quicker that participants assign stimuli to the grouping categories, the stronger their implicit bias towards the pairing (i.e., overweight & pleasant + thinness & unpleasant, or overweight & unpleasant + thinness & pleasant). Previous research has used the Implicit Association Test (IAT) to examine implicit bias towards people relating to social attitudes such as race, gender, and sexual orientation, as well as health and health-related behaviours such as exercise, drugs, and anxiety. The IAT is shown to have satisfactory internal consistency for the Implicit Association Test with Cronbach's alpha ranging from of 0.7 to 0.9.^25^^,^^26^

Questions with fixed-choice responses were posed to participants at various points throughout the webinar in real time, using the Zoom polling function (see Supplementary file). Participants were not shown the results of the polls on screen until the poll had closed to responses, to minimise response bias. All questions sought to establish participants’ perceptions and experiences of weight and non-weight focused clinical approaches within bariatric surgery services. Participants were also asked their perceived advantages and disadvantages of non-weight focused approaches to patient care in bariatric surgery services and what non-weight focused outcome measures they felt would be useful. Anonymous open-ended responses to these questions were collected using Padlet.^27^ Padlet is a real-time collaborative web platform where users can and share content to virtual bulletin boards.

### Data analysis

Data collected via BiasProof were analysed to identify whether HCPs have a bias in two directions; in this case, people considered overweight or thin. The BiasProof application uses the D algorithm, as recommended by Greenwald et al.,^28^ which produces the greatest internal validity of implicit association tests. The level of bias is assessed in line with IAT effects as a Cohen's d (standardised mean) for the whole sample.^29^ This is used to determine level of bias (i.e., no bias, slight, moderate, and strong in both directions).

A descriptive analysis of polling data and proposed alternative outcome measures were performed and expressed as frequency (%). Padlet responses were analysed using framework analysis, a systematic five stage process 1 = Familiarisation; 2 = Identifying a thematic framework; 3 = Indexing; 4 = Charting; and 5 = Mapping an interpretation.^30^ Two of the authors (S.A. - a dietitian, and E.S. - a clinical psychologist) independently read through participant responses (stage 1) and then met to develop an initial framework (stage 2). The data were then independently indexed and summarised (stage 3 and 4) and then together the authors (S.A. and E.S.) mapped and interpreted the data (stage 5). The concept map was then reviewed by one author (P.C. – a patient representative) as a method of researcher triangulation.^31^ Anonymised verbatim participant quotes were extracted to illustrate themes.

### Ethical approval

The study was approved by Coventry University Ethics Committee (P126611). All participants were asked to provide electronic written informed consent at the beginning of the webinar to contribute their data to the study.

### Role of funding source

Johnson & Johnson supported the educational event, funded the license cost of using the BiasProof Implicit Weight Bias Test and the open access article charge for publication. The funding source had no role in the conceptualisation of the study, analyses or interpretation and had no influence over the design and content of this article, or the decision to submit for publication.

## Results

### Study participants

A total of 113 HCPs attended the webinar, of which 73% (n = 82) consented to participate. Most participants who gave their profession stated they were dietitians (n = 33) or psychologists (n = 19) (Table 1). Due to the nature of live data collection during the webinar, the number of participants contributing data to each poll was variable.

**Table 1:** Profession of attendees.

Profession	Number who participated in polls
Dietitian	33
Psychologist	19
Nurse	3
Other	2
Did not answer	25

### Weight focused approaches within bariatric services

**Fig. 1:**
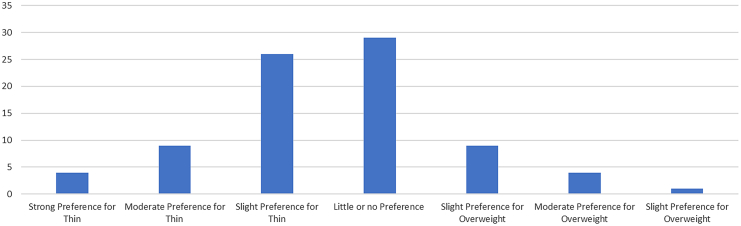
**Implicit weight bias preferences for thin and overweight**.

Over half of HCPs (51%, n = 42) had an implicit weight bias against PLwO (Fig. 1; participants with either a slight, moderate or strong implicit preference for thin), 53% (n = 31) indicated that their bariatric centre placed a mandate on the patient demonstrating weight loss to be listed for a bariatric surgery procedure, while 38% (n = 22) who responded indicated that a patient may be listed for surgery regardless of weight change. Meanwhile 62% (n = 39) of participants who responded disagreed or strongly disagreed that patients should be required to reach a weight loss target prior to being listed for bariatric surgery. Most (90%) HCPs agreed or strongly agreed that obesity services are too weight focused and that patients should not be weighed at every appointment (86%) (Table 2).

**Table 2:** Poll results.

Do you think bariatric surgery services are too weight focused? N = 60	Strongly agree % (n)	Agree % (n)	Neither agree or disagree % (n)	Disagree % (n)	Strongly disagree % (n)
	18% (11)	72% (43)	8% (5)	2% (1)	0% (0)

Does your service mandate weight loss in Tier 3 in order to be listed in bariatric services? N = 58	No – patient might be accepted regardless of weight change % (n)	Patients must demonstrate weight maintenance % (n)	Yes – the patient must demonstrate some weight loss (no specific target) % (n)	Yes – the patient must demonstrate 5% weight loss % (n)	Yes – the patient must demonstrate 10% weight loss % (n)
	38% (22)	9% (5)	15% (9)	36% (21)	2% (1)

Patient should be required to reach a weight loss target before being considered for bariatric surgery N = 63	Strongly agree % (n)	Agree % (n)	Neither agree or disagree % (n)	Disagree % (n)	Strongly disagree % (n)
	0% (0)	21% (13)	17% (11)	38% (24)	24% (15)

	Yes % (n)	No % (n)
Do you think we should be aiming to weigh patients at every contact within an obesity service? N = 71	14% (10)	86% (61)

### Perceived benefits of taking a non-weight focused approach within bariatric services

The benefits perceived by HCPs of taking a non-weight focused approach in bariatrics services were organised into three themes: 1) patient-led care 2) reducing stigma 3) supporting patient wellbeing. Direct anonymous participant quotes are provided in italics; given the anonymised nature of the data collection method we are unable to assign participant identification numbers to the quotes.

#### ‘People, not numbers’

HCPs felt that diverting focus away from a patient's weight would enable patient-led care, allowing the clinician to see the whole person by shifting focus away from numbers and allowing the patient to receive holistic care as well as have ownership of the direction of their care.

“This would take account of the individual, their personal circumstance, and their experiences”

“It’s about so much more than the numbers on the scales.”

“You get to focus on what the patients want to focus on …”

#### ‘Less shaming and blaming’

HCPs felt that moving away from a weight focus would have a positive impact on patients’ internalised shame and feelings of being subjected to judgement. In turn, this would have a positive impact on the patient–clinician relationship, whereby patients may be more open with their HCP.

“Helps patients be more honest and less judged”

“Moving away from the scales would promote better rapport and trust with a patient …”

“… potential to reduce shame associated with weight.”

“Makes the impact of change more tangible to daily life than a ‘good’ or ‘bad’ number.”

#### ‘Promoting quality and wellness’

HCPs felt that patients associated any weight gain with failure, discounting health benefits that may be present. Removing a focus from weight would enable patients to heal their relationship with self and consider quality of life improvements, instead.

“Patients are able to repair their relationship with food and their own sense of self without weight being a focus.”

“… may avoid patients' feelings of failure if they gain some weight even if the health benefits they are achieved are still evident.”

“Helps break the association of weight loss = good/perfect life and consider what changes will improve someone's quality of life more broadly.”

### Perceived barriers of taking a non-weight focused approach within bariatric services

Barriers to HCPs taking a non-weight focused approach were organised into themes of 1) loss of objectivity 2) inducing risk 3) difficulty demonstrating effectiveness.

#### ‘Fear of uncertainty’

HCPs felt that moving away from weight focus would mean alternative outcome measures would be needed and expressed that other outcome measures were more ‘abstract’ and therefore less useable within the clinical and commissioning context. HCPs also felt uncertain about what other alternative outcome measures were available.

“More abstract so harder to define objectively, harder to assess.”

“Other outcomes can be harder to measure, less objective.”

“Difficult to find other outcome measures to show what we are achieving.”

#### ‘Inducing risk’

There was tentativeness from HCPs about removing weight as a core focus in clinical practice. Using weight was seen as a way of enabling equity of care among patients. It was feared that without using weight, patients may have grounds to complain about subjectivity of care, with one patient being granted treatment over another. Weight was also relied upon as an anthropometric measure to identify malnutrition, and without using, there may be a clinical risk.

“Inequity – [using weight gives a] level playing field and no risk of complaints.”

“After bariatric surgery, the initial rate of weight loss along with dietary intake can be an indicator if the patient is losing weight too fast …”

#### ‘A need for proof’

HCPs felt obliged to demonstrate effectiveness to commissioners, patients, and to themselves. Weight change was seen as a surrogate result for behavioural changes implemented by patients. Ultimately, HCPs felt obliged for their practice to align with commissioners; and introducing a non-weight focused approach to commissioning may be a ‘battle’.

“Seeing weight going in the ‘right direction’ can help to motivate people.”

“If patients have engaged in our programme and put it into practice they should have lost weight.”

“Commissioners would require some sort of measure to evaluate the service.”

### Proposed alternative non-weight focused measures

Using non-weight focused outcome measures was seen by participants as an opportunity to celebrate the patient's *“non-scale victories”*. The most frequently suggested alternative non-weight focused measures were severity of co-morbidities (n = 9), quality of life (n = 6) and mental health (n = 6). However, some HCPs (n = 2) still suggested body composition should be measured in some way, if not by weight (Table 3).

**Table 3:** Proposed alternative non-weight focused clinical outcome measures.

	n=
Improvement in co-morbidities	9
Quality of life	6
Mental health	6
Able to undertake activities of daily living	4
Non-specific ‘patient-centred’ goals	3
Dietary quality	3
Cardiovascular fitness	2
Attendance	1
Physical activity levels	1
Values	1
Energy levels	1
Disordered eating	1
Body composition	2

## Discussion

HCPs’ interactions with patients can be affected by implicit bias and ultimately this can impact on quality of care and the patient-HCP relationship. Until our present study, there was little published data on the prevalence on weight bias among HCPs specialising in obesity. We have reported data for the first time on implicit weight bias among HCPs working in bariatric surgery services, finding that just over half of HCPs had an implicit weight bias against PLwO.

Comparing our findings to the few studies that have reported implicit weight bias prevalence among HCPs, our findings suggest that implicit weight bias is less prevalent among bariatric surgery HCPs, compared with HCPs who work outside of obesity services. In our present study, 51% of HCPs exhibited implicit weight bias with 16% having a moderate or strong implicit weight bias. In US populations, the overall prevalence of exhibited implicit weight bias (slight, moderate, or strong) amongst physicians and medical students is reported to range between 74 and 87%.^32^^,^^33^ Most had a moderate or strong implicit weight bias (59–65%).^32^^,^^33^ However, it should be noted that none of the participants in our study were medically trained HCPs (i.e., physicians or surgeons) and therefore our findings are not directly comparable.

Nearly two-thirds (63%) of our participants were dietitians or psychologists. There is no published research investigating implicit weight bias among psychologists and only two studies have reported prevalence of implicit weight bias among dietitians.^34^^,^^35^ In the US, 76.0% of dietitians had strong or moderate implicit weight bias^34^ while in Germany, prevalence of moderate or strong implicit weight bias was lower, between 56.5 and 61.7%.^35^

We also examined weight focused approaches taken in UK bariatric surgery services. In similarity with data from a national UK survey also undertaken in 2021,^15^ we found that more than half of bariatric surgery services required a weight loss target to be met.^15^ This contrasts with the reality of clinical practice, as only one in five HCPs in our study agreed that patients should be mandated to lose weight before being approved for bariatric surgery.

The proportion of services mandating weight loss in the UK is higher than internationally. In a survey of worldwide clinical practice across 76 countries,^16^ 43.9% of HCPs indicated that mandatory weight loss was required for all patients. We did not examine the reasons why, however internationally the most frequent reasons provided for mandating weight loss were to “assess patient's motivation for surgery” and to “improve weight loss outcomes”.^16^

Mandating weight loss to improve weight loss outcomes after bariatric surgery is not evidence-based practice. Evidence from a multi-centre cohort study in the UK showed that weight loss prior to bariatric surgery does not predict weight loss after bariatric surgery,^36^ supporting that obesity is driven by biology and not by a lack of “motivation”. This is supported by Dixon et al.^37^ who showed that weight loss outcomes after bariatric surgery had no association with patients’ readiness to change. By mandating weight loss, bariatric services in the UK are contradicting clinical guidance from NICE,^38^ where bariatric surgery is recommended when “all appropriate non-surgical measures have been tried but the person has not achieved or maintained adequate, clinically beneficial weight loss”. Therefore, the practice of mandating weight loss, as reported by over half of our participants, reinforces beliefs about weight controllability and incites blame.^14^ It is therefore arguable that mandating weight loss is, in itself, stigmatising. As consequence, PLwO are being denied access to bariatric surgery by the very HCPs whose role it is to provide treatment for their obesity.

Nearly all HCPs in our study agreed that services are too weight focused (90%) and most agreed that patients should not be weighed at every appointment (86%). Our qualitative analysis explored this in more depth. HCPs felt that assessing treatment outcomes is about *“so much more than the number on the scales”*. HCPs perceived those benefits of taking a non-weight focused approach would be a reduction in stigma (‘less shaming and blaming’), enablement of patient-led care (‘people, not numbers’) and enhancement of patient wellbeing (‘promoting quality and wellness’).

Likewise, in a review of strategies to address weight stigma in healthcare, it is recommended that HCPs should move away from a weight-centric approach, towards an approach focused on health and weight-inclusivity.^13^ Positive treatment outcomes beyond weight, referred to as *“non-scale victories”*, were proposed by HCPs in our study. The most frequently suggested measures were severity of co-morbidities, quality of life, and mental health, which suggests HCPs value outcome measures of health improvements. In the literature, weight-inclusive programmes that focus on the social experience of living with obesity and psychological aspects of eating have been shown to provide physical and psychological benefit to patients.^39^, ^40^, ^41^

Previous attempts to reduce weight stigma have largely either been ineffective or, where there has been a reduction in stigma, these have been slight with effects dissipating over time. Intervention attempts include improved education about obesity and weight-related health amongst HCPs and trainee HCPs,^42^^,^^43^ raising awareness and addressing beliefs about the controllability of obesity,^44^, ^45^, ^46^ evoking empathy,^47^^,^^48^ and combining weight inclusive approaches with raising awareness of weight bias.^49^ Given the pervasiveness of weight stigma and discrimination, not only in healthcare, but across society, there is a need to address weight stigma across all levels from policy to practice. Indeed, the formation of weight stigma attitudes and beliefs amongst HCPs is likely have started before their professional role, and may be reinforced outside of healthcare.

HCPs also thematically cited barriers to adopting a non-weight focused approach within bariatric surgery services. These were losing objectivity (‘fear of uncertainty’), ‘inducing risk’ and difficulty to demonstrate effectiveness to patients, commissioners and to the HCP (‘a need for proof’).

The receipt of a complaint can has a negative impact on the emotional state of the HCP^50^ and, naturally, HCPs seek to avoid complaints as means to protect their psychological wellbeing. HCPs in our study were concerned that making decisions about patient care without the objectivity of weight, would induce a greater likelihood of complaints. Although there is no published literature on patient complaints relating to obesity services specifically, previous research has found that certain specialities are more likely to attract complaints; particularly fields where a patient's perception of their body image may be a particularly emotive subject.^51^

Moreover, HCPs felt that moving focus away from weight as a part of routine assessment may incite clinical risk. There is a strong positive correlation between weight loss and lean body mass loss following bariatric surgery.^52^ Therefore, if regular weight measurements are not obtained, patients at risk of sarcopenia may not receive a more intensive level of support and monitoring, to minimise the extent of skeletal muscle loss.^53^^,^^54^

On the other hand, HCPs also felt that where the patient's weight is *“going in the right direction”*, a weight-focused approach supports effective clinical treatment by providing positive feedback to improve a patient's motivation. While self-weighing has been shown to be an effective tool for weight loss,^53^ there is paucity of research to examine whether being weighed by HCPs is clinically effective. Prior research found that patients attending weight management programmes felt a sense of obligation to not “let down” their HCP.^54^ Consequentially, being weighed in healthcare settings has the potential to invoke internalised shame and distress for PLwO.

HCPs felt that if services were to move away from a weight-focused approach, weight would need to be replaced with another measure to enable HCPs to *“show what we are achieving”* but felt it would be *“difficult”* to find an alternate outcome measure. The BARIACT core outcome set does describe recommended measures in the reporting of bariatric surgery research studies.^55^ Alongside weight, non-weight focused outcome measures are recommended, including health-related outcomes of “diabetes status” and “cardiovascular risk”, and “overall quality of life”, as a patient-reported outcome. However, so far, there is not a published core outcome set for bariatric surgery clinical practice. Hence, if bariatric surgery HCPs are to feel able to shift focus away from weight; a core outcome set is needed that recommends alternative time-efficient and validated outcome measures that are suited to the pragmatism of clinical practice.

This study has several key strengths. For the first time, we have reported data on implicit weight bias among HCPs working in bariatric surgery services. We also provide data on implicit weight bias amongst non-medical HCPs, including dietitians and psychologists, and therefore this study offers a novel insight into HCP groups that have been under-represented previously in research. Using the BiasProof application enabled the automatic, unconscious bias to be measured.^56^ Data collection was entirely anonymous, allowing participants to be open with their responses and therefore overcoming, to some extent, a potential social desirability bias,^57^ enhancing the internal validity of the findings. Given the exploratory nature of our study, we took a mixed methods approach; using both quantitative and qualitative methodologies to gather participants attitudes and current practice. We used researcher triangulation in the qualitative analysis, offering different perspectives on the phenomenon of interest^58^ from a psychologist, dietitian, and a patient representative with lived experience of being treated within a bariatric surgery service, thereby enhancing the credibility of the findings.

There are also limitations to our study. Participants were recruited via convenience sampling and 27% of webinar delegates did not consent for their data to be used, representing a non-responder bias. The study population were predominantly dietitians and psychologists and therefore our findings may not be generalisable across all professions working in bariatric surgery services. It is also possible that, although the webinar was aimed at bariatric surgery HCPs, some participants may not have worked in bariatric surgery services. The nature of data collection within a live webinar event also meant that there was variability in the extent of missing data across the poll questions. Collecting qualitative data in electronic written form, rather than verbally, will have impacted on the richness of the data.^59^

Identifying widely applicable ways to effectively reduce healthcare related weight stigma is urgently needed.^13^ One of many approaches could be to adopt non-weight focused approaches within obesity services. However, given the barriers cited by HCPs in our study, alternative outcome measures need to be validated and the acceptability of these within clinical practice and with clinical commissioners will need to be examined.

While we have a strong ethical argument to address weight stigma in healthcare, we need rigorous empirical research into specific interventions to reduce implicit weight bias among HCPs and the future generation. Prior research has found that the strongest predictor of implicit weight bias is younger age.^20^ From meta-analysis data, we also know that the malleability of weight bias is more profound, although not statistically significant, among healthcare students compared to qualified HCPs.^60^ This indicates that the prime opportunity to target interventions for implicit weight bias is within healthcare training in the educational setting,^13^ where healthcare students may still be forming their attitudes and beliefs toward PLwO.

In conclusion, HCPs’ interactions with patients can be affected by implicit bias and ultimately impact quality of care and the patient-HCP relationship. We found that one in two HCPs who work with patients with the most clinically severe obesity, exhibit an implicit weight bias against PLwO. Our findings support the need to develop targeted interventions for HCPs to address implicit weight bias and the necessity for future robust research into alternative non-weight focused outcome measures.

## Contributors

S.A. - conceptualisation, data curation, formal analysis, funding acquisition, investigation, methodology, project administration, validation, visualisation, writing - original draft, writing - review and editing. E.S. - formal analysis, investigation, validation, visualisations, writing - review and editing. H.M.P. – data curation, formal analysis, investigation, validation, visualisation, writing - review and editing. P.C. - validation, visualisation, writing - review and editing. S.W.F. - data curation, formal analysis, investigation, methodology, software, validation, visualisation, writing - original draft, writing - review and editing.

## Data sharing statement

Restrictions due to informed consent apply to the availability of these data.

## Declaration of interests

S.A. has received speaker honorarium from Johnson & Johnson for educational events and support for attendance at academic meetings from Novo Nordisk and British Dietetic Association GET Fund. S.A. reports research grants from British Dietetic Association Obesity Specialist Group. E.S. has received speaker honorarium from Johnson & Johnson for educational events. P.C. has received speaker honorarium from Johnson & Johnson for educational events. S.W.F. reports research grants from National Institute for Health Research, Public Health England, Doncaster Council, West Yorkshire Combined Authority, Johnson and Johnson, Novo Nordisk and the University of Leeds, personal fees from the Royal College of General Practitioners, Institutional fees from Public Health England, and support for attendance at academic meetings from Novo Nordisk and Johnson & Johnson. H.M.P. has received speaker honoraria from Johnson & Johnson and Novo Nordisk for educational events. Honoraria received for participating in the development of an algorithm for the management of obesity in primary care supported by arm's length sponsorship from Novo Nordisk. Co-author on a publication of UK data from a study funded by Novo Nordisk (no honorarium). H.M.P. reports research grants from National Institute for Health Research, Public Health England and the Office for Health Improvement and Disparities.

## Appendix A Supplementary data

Supplementary File 1

Caption for the supplementary material
