# Supplementary material for: “Is it time to throw out the weighing scales?” Implicit weight bias among healthcare professionals working in bariatric surgery services and their attitude towards non-weight focused approaches
Source: eClinicalMedicine. 2022 Dec 14;55:101770. doi: 10.1016/j.eclinm.2022.101770 (PMC9772809; doi:10.1016/j.eclinm.2022.101770)
Supplement: Supplementary File 1 [file mmc1.docx]

**Supplementary file 1: Poll questions and fixed responses**

1. Do you think bariatric surgery services are too weight focused?
   1. Strongly Agree
   2. Agree
   3. Neither Agree or disagree
   4. Disagree
   5. Strongly disagree
2. Do you think we should be aiming to weigh patients at every contact within an obesity service?
   1. Yes
   2. No
3. Does your service or the service you refer into to mandate weight loss in Tier 3 in order to be listed in bariatric services:
   1. No – patient might be accepted regardless of weight change
   2. Patients must demonstrate weight maintenance
   3. Yes – the patient must demonstrate some weight loss (no specific target)
   4. Yes – the patient must demonstrate 5% weight loss
   5. Yes – the patient must demonstrate 10% weight loss
4. Patients should be required to reach a weight loss target before being considered for bariatric surgery:
5. Strongly Agree
6. Agree
7. Neither Agree or disagree
8. Disagree
9. Strongly disagree
