# Supplementary material for: “Is it time to throw out the weighing scales?” Implicit weight bias among healthcare professionals working in bariatric surgery services and their attitude towards non-weight focused approaches
Source: eClinicalMedicine. 2022 Dec 14;55:101770. doi: 10.1016/j.eclinm.2022.101770 (PMC9772809; doi:10.1016/j.eclinm.2022.101770)
Supplement: Caption for the supplementary material [file mmc2.docx]

**Caption for the supplementary material**

Supplement 1 – Poll questions and fixed responses
